# The Pharmacodynamics Study of Insect Defensin DLP4 Against Toxigenic *Staphylococcus hyicus* ACCC 61734 *in Vitro* and *Vivo*

**DOI:** 10.3389/fcimb.2021.638598

**Published:** 2021-05-05

**Authors:** Xuanxuan Ma, Na Yang, Ruoyu Mao, Ya Hao, Xue Yan, Da Teng, Jianhua Wang

**Affiliations:** ^1^ Gene Engineering Laboratory, Feed Research Institute, Chinese Academy of Agricultural Sciences, Beijing, China; ^2^ Key Laboratory of Feed Biotechnology, Ministry of Agriculture and Rural Affairs, Beijing, China; ^3^ New Hope Liuhe Co., Ltd., Quality Control for Feed and Products of Livestock and Poultry Key Laboratory of Sichuan Province, Chengdu, China

**Keywords:** *S. hyicus*, insect defensin DLP4, *in vitro* antibacterial activity, antibacterial mechanism, *in vivo* therapeutic effect

## Abstract

*Staphylococcus hyicus* (*S. hyicus*), as the main pathogen of exudative epidermitis (EE) in piglet, can cause a wide variety of diseases, ranging from bovine mastitis, chicken arthritis and even human sepsis, which has brought serious threats to animals and human. The potential threat of *S. hyicus* infection to both public and animal health has aroused great concern. The aim of our study was to explore the efficacy of insect defensin DLP4 against *S. hyicus* ACCC 61734 *in vitro* and *in vivo*. The *in vitro* efficacies of DLP4 against *S. hyicus* ACCC 61734 showed high antibacterial activity (0.92 μM), a long postantibiotic effect (9.54 h), a synergistic effect with ceftriaxone, penicillin and amoxicillin, a stable bacteriostatic effect, and intracellular bacteriostatic activity against *S. hyicus* ACCC 61734 in HaCaT cells. Besides, the antibacterial mechanism of DLP4 against *S. hyicus* ACCC 61734 was explored for the first time, which indicated that the antibacterial effect of DLP4 was related to its ability to destroy cell wall and generate membrane vesicles. The *in vivo* therapeutic effect of DLP4 was evaluated through mouse abscess model, and the results showed that DLP4 could effectively alleviate the mouse skin abscess by inhibiting bacterial proliferation and regulating cytokines. This study first demonstrated that DLP4 may be a promising therapeutic agent against *S. hyicus* ACCC 61734 infection.

## Introduction

Exudative epidermitis (EE), also known as “greasy pig disease”, is an occasional and contagious skin disease, which predominantly infects weaning and newborn piglet (Olivry and Linder, 2009). *Staphylococcus hyicus* was widely acknowledged as the major causative agent of EE, it can produce up to six various 27–30 KDa exfoliation toxins (*ExhA*, *ExhB*, *ExhC*, *ExhD*, *ShetA*, and *ShetB*), which was always considered as the key pathogenic factor of EE (Andresen et al., 1993; Andresen and Ahrens, 2004). Previous studies have revealed that the four *Exh* exfoliation toxins and *ShetB* belong to serine proteases family, whose amino acid sequences are highly homologous with the exfoliative toxins (*ETA*, *ETB*, and *ETD)* of *Staphylococcus aureus*, especially in the vicinity of the active sites. Besides, like exfoliative toxins (ETs), *Exh* can specifically recognize and digest desmoglein 1 (Dsg1) (Fudaba et al., 2005; Nishifuji et al., 2005), causing the separation of cells in the upper stratum spinosum, forming typical symptoms of dermatitis, these symptoms all indicated that they have similar pathophysiological functions in animals and human (Hanakawa et al., 2002; Nishifuji et al., 2008; Mariutti et al., 2015). Recently, the outbreak of EE in worldwide has brought huge economic losses to the breeding industry (Arsenakis et al., 2018), and the prevention and treatment of EE have increasingly attracted widespread attention.

Presently, with the wide-ranging inappropriate use of antibiotics, multidrug resistant *S. hyicus* has been isolated from piglets around the world (Holmer et al., 2019). Previous tests showed that among 142 strains isolated from Canada, the strains with penicillin G, ampicillin, ceftiofur, and tetracycline resistance accounted for 90, 90, 70, and 55.6%, respectively (Park et al., 2013), and the strains isolated from Korean developed remarkable resistance to amoxicillin, lincomycin, penicillin, streptomycin, and tetracycline (Park et al., 2018). Besides, the isolated *S. hyicus* ACCC 61734 has also been proved to develop resistance to penicillin, sulfamethoxazole, chloramphenicol, streptomycin, azithromycin, erythromycin, tetracycline, bacitracin and norfloxacin in our previous study (Ma et al., 2019). Given the increasing acquisition of antibiotic resistance by pathogens, the prevention and control of *S. hyicus* infection have been a huge challenge, which indicates an urgent need for new effective antibacterial drugs. Antimicrobial peptides (AMPs), as a critical part of the natural defense system, are small cationic peptides with extensive antibacterial activity and have been considered as an alternative to traditional antimicrobial agents for their high antibacterial activity and low resistance (Hancock and Sahl, 2006). According to our previous study, DLP4, as an insect defensin from *Hermetia illucens*, presented a broad-spectrum antibacterial activity against gram-positive bacteria, and had no significant hemolytic activity to mice erythrocytes (1.46% at 512 µg/ml) or moderate cytotoxicity to mouse peritoneal macrophages (17.39% at 128 µg/ml). Meanwhile, DLP4 was difficult to develop resistance due to its unique antibacterial mechanism (Li et al., 2017). All these results indicated that DLP4 may be considered to be a promising drug for treating *S. hyicus* infections.

Based on our previous research on the antibacterial effects of DLP4 on *S. aureus*, this study systematically explored the *in vitro* antibacterial properties and mechanisms of DLP4 against *S. hyicus* ACCC 61734. In addition, the *in vivo* efficacies of DLP4 were confirmed using mouse abscess model caused by *S. hyicus* ACCC 61734.

## Materials and Methods

### Materials

*S. hyicus* NCTC 10350 was purchased from National Collection of Type Culture (NCTC). Clinical strain *S. hyicus* ACCC 61734 was isolated from the kidney of a piglet with defined EE by Tianjin Animal Science and Veterinary Research Institute. DLP4 was prepared as described in our previous study (Li et al., 2017). Six-week-old female BALB/c mice (SPF) were purchased from the Vital River Laboratories (VRL, Beijing, China). All other chemical reagents were of analytical grade. All animal researches have been approved by the Animal Care and Use Committee of the Feed Research Institute of the Chinese Academy of Agricultural Sciences (CAAS).

### Antibacterial Assay *In Vitro*

#### Antimicrobial Activity Determination

The minimum inhibitory concentrations (MICs) of DLP4 against *S. hyicus* ACCC 61734 and *S. hyicus* NCTC 10350 were determined using the microtiter broth dilution method described previously (Zhang et al., 2014). The mid-log phase bacteria were diluted to 1 × 10^5^ CFU/ml with fresh MHB medium, a total of 90 μl of cell suspension and 10 μl of serially twofold diluted peptide with the final concentrations of 0.0625–128 μg/ml were added into 96-well plates. The plates were incubated for 18–24 h at 37°C. The MIC value was determined as the lowest peptide concentration at which no bacterial growth was observed. Ceftriaxone, ceftiofur, ofloxacin, and amoxicillin were used as controls. All assays were performed in triplicate.

#### Growth Kinetic Assays

Growth kinetic determination was performed in order to estimate the pharmacodynamics of DLP4 against *S. hyicus* ACCC 61734. The tested strains were diluted to 10^5^ CFU/ml and mixed with different concentrations of peptide at final peptide concentrations of 1×, 2×, or 4× MIC, respectively, and then were incubated at 37°C. After 0, 0.5, 1, 2, 4, 8, 10, 12, and 24 h incubation, the samples (100 µl) were taken for colony counting on MHA plates. Ceftriaxone and 0.9% NaCl were used as positive and blank controls, respectively (Zhang et al., 2014).

#### The Postantibiotic Effect (PAE) of DLP4 Against *S. hyicus*

PAE assays of DLP4 against *S. hyicus* ACCC 61734 were manipulated and calculated as previously described (Zhao et al., 2019). The specific steps are described as follows: (1) PAE induction stage: Tested strain *S. hyicus* ACCC 61734 was diluted to 10^8^ CFU/ml and mixed with corresponding concentration of DLP4 at final concentrations of 1×, 2×, or 4× MIC, respectively, then were incubated at 37°C for 2 h, 2× MIC ceftriaxone and PBS were used as positive and blank control, respectively; (2) Drug removal and reconstruction: each of the above systems was diluted 1,000 times to remove the drug and reconstruct the growth system, which was defined at this point as the 0 h point after reconstruction; (3) Colony counting: the above reconstructed system was incubated at 37°C, and the bacterial colonies were counted at 0, 2, 4, 6, 8, 10, 12, 20, 22, and 24 h by sampling gradient dilutions, respectively; (4) PAE calculation: PAE (h) = T − C, T: time required for the number of colonies in the drug-treated group to be 10 times as the number of colonies at 0 h, C: corresponding time required for the blank control group.

#### Synergistic Interactions of DLP4 With Other Antibiotics

The possible synergistic effects of DLP4 in combination with penicillin, ceftriaxone, amoxicillin and ofloxacin were performed using the checkerboard method. The specific steps are described as follows: An 80 μl of *S. hyicus* ACCC 61734 (10^8^ CFU/ml) was added into the 96-well plate, and then 10 μl of DLP4 and antibiotics were added into the above 96-well plate at a final concentration of 1/16 to 8 × MIC in the horizontal and vertical directions, respectively. Finally, the 96-well plate was incubated at 37°C for 18 h. The optimal drug combination and concentration was determined based on the FICI. FICI determination criteria: FICI ≥4: antagonistic effect, 1 < FICI ≤4: irrelevant effect; 0.5 < FICI ≤1: additive effect; and FICI ≤0.5: synergistic effect (Zheng et al., 2017).

#### Resistance Induction Test

To speculate whether the long-term use of antimicrobial agents could cause the bacteria to develop corresponding drug resistance, *S. hyicus* ACCC 61734 cells were exposed to sub-MIC concentration of DLP4 or ceftriaxone, and incubated for 30 d, then used for continuous MIC measurement (Narayana et al., 2015; Li et al., 2017).

#### Intracellular Bacteriostatic Efficacy

To investigate the intracellular antibacterial effect of DLP4, the invasion assay was firstly operated as described previously (Wang et al., 2018b). In brief, HaCaT cells (2.5 × 10^5^ cells, 750 μl) were inoculated into 12-well, after incubation for 24 h in minimum Eagle's medium (MEM) with 10% FBS (without antibiotics), The mid-log phase *S. hyicus* ACCC 61734 cells were added into the plates at a concentration of 2.5 × 10^7^ CFU/ml in MEM with 10% FBS (without antibiotic) and incubated for 30 min, gentamicin (100 μg/ml) was added and incubated for 2 h to remove extracellular bacteria. After washing with PBS, HaCaT cells were fixed in 2.5% glutaraldehyde overnight at 4°C, postfixed with 1% osmium tetroxide (OsO_4_), dehydrated in a gradient ethanol series, then cells were immersed in epoxy resin; the ultramicrotome was used to acquire the thin sections, followed by staining with 1% uranyl acetate. Images were visualized by a TEM (JEM1400, JEDL, Japan); (2) HaCaT cells were treated with different concentrations of DLP4 (5×, 10×, and 50× MIC) for 24 h, washed again and lysed with Hanks buffered saline solution (0.1% bovine serum albumin and 0.1% Triton-X). The numbers of intracellular bacteria were measured at 0 and 24 h by colony counting.

### The Antibacterial Mechanism of DLP4

#### Membrane Permeabilization Assays

The influence of DLP4 on *S. hyicus* ACCC 61734 cell membrane was determined using the DNA intercalating dye propidium iodide (PI) uptake assay by flow cytometry (Zheng et al., 2017). *S. hyicus* ACCC 61734 cells in mid-log phase (1 × 10^8^ CFU/ml) were treated with DLP4 (1×, 2×, and 4× MIC) at 37°C for 5, 30 and 120 min, respectively. After staining with PI, the samples were analyzed by a Flow Cytometer (FACS Calibur, BD, USA).

#### Electron Microscopy Observations

Mid-log phase *S. hyicus* ACCC 61734 cells, were treated with 4× MIC DLP4 for 2 h at 37°C. Subsequently, the samples were fixed with 2.5% glutaraldehyde overnight at 4°C, dehydrated in a gradient ethanol series, and dried with CO_2_. Finally, the samples were sputtered using gold-palladium and observed under a QUANTA200 scanning electron microscope (SEM), (FEI, Philips, Netherlands) (Hao et al., 2017).

Transmission electron microscopy (TEM) was used for further observations of the cell morphology and intracellular changes. Bacterial samples were treated using the same method as described above (Hao et al., 2017). After postfixation with 1% OsO_4_ and gradient dehydration, cells were embedded in resin, sliced, and placed on a formvar carrier grid, followed by uranyl acetate and lead citrate treatments. Microscopy was performed using a TEM (JEM1400, JEDL, Japan).

### Mouse *In Vivo* Test

To evaluate the *in vivo* efficacy of DLP4, the mouse abscess model was established. Six-week-old female BALB/c mice were randomly allocated into four groups (20 mice/group) including blank control (uninfected and untreated), negative control (infected but untreated) and two treated group (infected and treated with DLP4 and ceftriaxone, respectively). All mice were anesthetized and shaved on the abdomen. Next, the mice were subcutaneously injected with 200 μl of the exponential phase *S. hyicus* ACCC 61734 (2.5 × 10^9^ CFU/ml) (Ding et al., 2012; Wang et al., 2018a), after infection for 24 h, DLP4 (150 μg/flank) or ceftriaxone (600 μg/flank) was injected subcutaneously near the abdomen once a day for 3 d. During the period of treatment, the clinical wound sites were imaged and further measured by Image J to record abscess area. After infection for 3, 7, and 12 d, mice were euthanized and skin tissue samples were collected, weighed and homogenized, then the suspension of abscesses was serially diluted in sterile PBS for colony counting. Additionally, the levels of inflammatory factors interleukin-6 (IL-6) and tumor necrosis factor-α (TNF-α), and epidermal growth factor (EGF) released from abscesses were measured by ELISA kit. At the same time, the abscess samples of mice at days 3 and 12 were fixed in 4% paraformaldehyde at 4°C for 24 h; stained with hematoxylin eosin (HE) and observed by a light microscope (OLYMPUS BX43).

### Statistical Analysis

All data were presented as the means ± standard deviation (SD) and all statistical analyses were performed using GraphPad Prism software v8.0 (GraphPad Software, USA). Statistical significance of groups was analyzed using the one-way ANOVA and Tukey multiple comparison.

## Results

### *In Vitro* Antibacterial Activity

#### MIC Assays

As shown in **Table 1**, DLP4 showed the antibacterial activity against *S. hyicus* ACCC 61734 and *S. hyicus* NCTC 10350 with the MIC values of 0.92 μM. However, the tested antibiotics displayed low activity against *S. hyicus* ACCC 61734 and *S. hyicus* NCTC 10350, such as ceftriaxone with the MIC values over 3.02 μM and ceftiofur with the MIC values over 12.08 μM, which were far higher than those of DLP4. The results indicated that DLP4, as the promising antimicrobial agent, displayed more potent antibacterial activity against *S. hyicus*.

**Table 1:** MIC assay of DLP4 and antibacterial agents against *S. hyicus*.

Antibacterial Agents	*S. hyicus* NCTC10350		*S. hyicus* ACCC 61734	
	μM	μg/ml	μM	μg/ml
DLP4	0.92	4	0.92	4
Ceftriaxone	3.02	2	6.04	4
Ceftiofur	12.08	8	24.16	16
Ofloxacin	5.38	2	43.2	16
Amoxicillin	2.38	1	2.38	1
Penicillin	5.98	2	2.99	1

The unit of μM means drug molar concentration; the unit of μg/ml means drug mass concentration.

#### Bactericidal Kinetics

**Figure 1:**
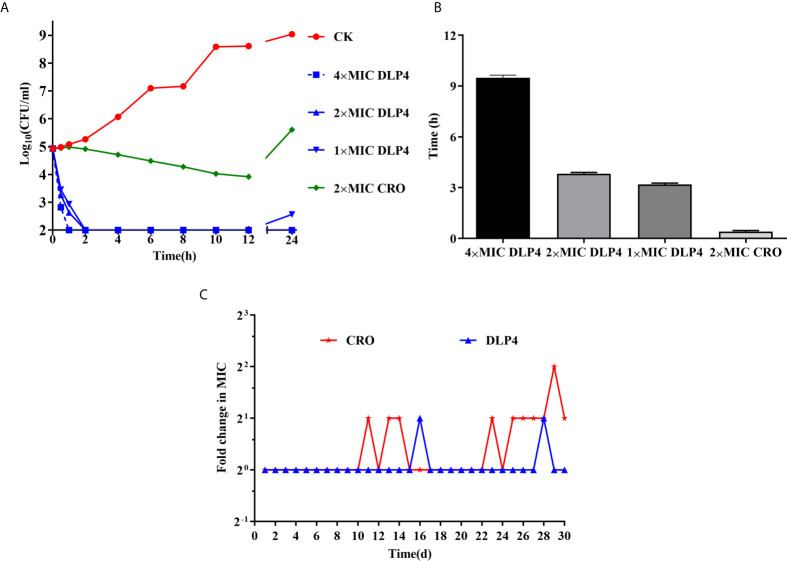
*In vitro* antibacterial activity. **(A)** Time-kill curves of peptides and antibiotics against the *S. hyicus* ACCC 61734 *in vitro*. CK: *S. hyicus* ACCC 61734 was cultivated in culture medium without any drugs. Three duplicate observations were made, results were given as mean ± SD (n = 3). **(B)** The PAE (h) of different doses of drugs. Results were given as mean ± SD (n = 3). **(C)** Drug resistance evolution of *S. hyicus* cultured with sub-MIC DLP4 and ceftriaxone.

The bactericidal kinetics of DLP4 against *S. hyicus* ACCC 61734 were shown in **Figure 1A**. In the blank control group, the *S. hyicus* cells reached 8.6 Log_10_ CFU/ml after cultured for 12 h, and then entered into stationary phase. However, *S. hyicus* ACCC 61734 cells decreased dramatically after treatment with DLP4, and the bacteria were inhibited completely after treatment with 4× MIC DLP4 for 1 h and 2× MIC DLP4 for 2 h, and the bacteria were temporarily inhibited after treatment with 1× MIC DLP4 for 2 h but regrew after 12 h, which demonstrated that DLP4 has dose-dependent growth inhibition against *S. hyicus* ACCC 61734. In addition, the bactericidal rate of ceftriaxone (2× MIC) apparently lagged, reduced slowly and even rebounded after incubation for 12 h. These results indicated that DLP4 had the advantages of a rapid and non-rebounding activity compared with ceftriaxone.

#### PAE

As shown in **Figure 1B**, the PAE of DLP4 against *S. hyicus* ACCC 61734 was evaluated. The PAE values of DLP4 against *S. hyicus* ACCC 61734 were 9.54, 3.81, and 3.23 h at 4, 2, and 1× MIC, respectively, which were 28.9, 11.5, and 9.78 times longer than that of 2× MIC ceftriaxone (0.33 h), which indicated that the PAE effect of DLP4 was in a concentration dependent manner. The results indicated that DLP4 displayed a longer influence on the growth of *S. hyicus* ACCC 61734 than ceftriaxone.

#### Synergy With Antibiotics

The influence on efficiency of DLP4 in combination with several common antibiotics compared with their own individual activities was determined by FICI values. As shown in **Table 2**, the FICI values of combination of DLP4 with the tested antibiotics ceftriaxone, penicillin, amoxicillin and ofloxacin were 0.3125, 0.3125, 0.3725, and 0.625, respectively. According to the synergy index, the results showed that DLP4 displayed a synergy effect against *S. hyicus* when combined with ceftriaxone, penicillin and amoxicillin, and an additive effect when combined with ofloxacin. There was no indifference or antagonism, which may be due to the differences in antibacterial mechanisms between DLP4 and their antibacterial mechanisms were non-interfering each other.

**Table 2:** The FICI of each combination of different antibiotics with DLP4.

S. hyicus ACCC 61734					
Combination	Variety	MIC^a^ (μg/ml)	MIC^c^ (μg/ml)	FIC	FICI
DLP4-CRO	DLP4	4	1	0.25	0.3125^a^
	CRO	4	0.25	0.0625	
DLP4-AMX	DLP4	4	0.5	0.125	0.375^a^
	AMX	1	0.25	0.25	
DLP4-PEN	DLP4	4	1	0.25	0.3125^a^
	PEN	1	0.0625	0.0625	
DLP4-OFX	DLP4	4	0.5	0.125	0.625^b^
	OFX	16	8	0.5	

MIC^a^ indicates the MIC of drug used alone; MIC^c^ indicates the MIC of drug used in combination;

^a^Synergic effect; ^b^Additive effect.

#### Resistance Induction Test

As shown in **Figure 1C**, after 30 serial passages exposed to antibacterial drugs, the MIC of DLP4 against *S. hyicus* ACCC 61734 remained unchanged. While the MIC of ceftriaxone increased two times. These results displayed that both ceftriaxone and DLP4 are stable and not easy to develop drug resistance against *S. hyicus* ACCC 61734.

#### Intracellular Bacteriostatic Efficacy

**Figure 2:**
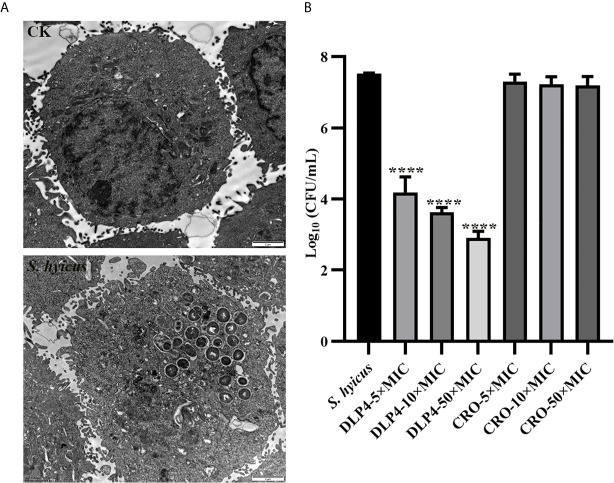
Intracellular bacteriostatic efficacy of DLP4. **(A)** Morphologies of *S. hyicus* ACCC 61734 in HaCaT cells; **(B)** Intracellular antibacterial activity against *S. hyicus* ACCC 61734, results were given as mean ± SD (n = 3). All data were analyzed by the one-way ANOVA and Bonferroni multiple comparison. ****P < 0.0001.

As shown in **Figure 2A**, *S. hyicus* ACCC 61734 could successfully enter into HaCaT cells without destroying the cell membrane, and mainly located in the small vacuoles (tight phagosomes). At the same time, the bacteria were in a state of division, indicating that the bacteria could reproduce normally in the HaCaT cell, which was consistent with the previous results of *S. aureus* (Wang et al., 2018b). Meanwhile, the intracellular sterilization assay showed that DLP4 could enter into HaCaT cells and effectively kill *S. hyicus* ACCC 61734. Compared with the negative control group, the intracellular bactericidal rate after DLP4 treatment reached up to 99.9% (from 7.5 Log_10_ CFU/ml to 3.6 Log_10_ CFU/ml) (**Figure 2B**). And the results also showed that ceftriaxone did not kill the bacteria in HaCaT cells as it can’t enter into cells.

### The Antibacterial Mechanism of DLP4

#### Integrity of Bacterial Membrane

**Figure 3:**
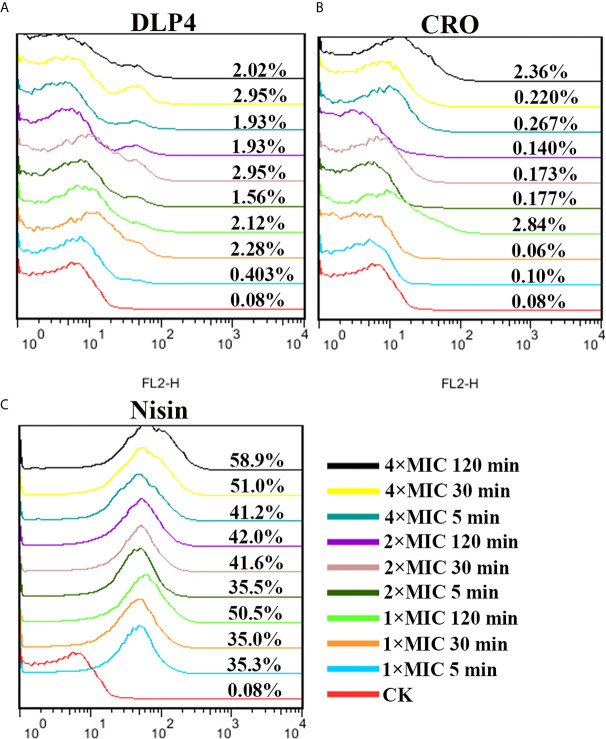
Analysis of PI-staining in *S. hyicus* ACCC 61734 treated with DLP4. **(A)** Analysis of PI-staining in *S. hyicus* ACCC 61734 treated with DLP4. **(A)** Pathogen treated with 1×, 2× and 4× MIC DLP4 for 5, 30 and 120 min, respectively; **(B)** Pathogen treated with 1×, 2× and 4× MIC CRO for 5, 30 and 120 min, respectively; **(C)** Pathogen treated with 1×, 2× and 4× MIC Nisin for 5, 30 and 120 min, respectively.

Based on its ability to permeate damaged cell membranes and insert into nucleic acid, PI staining was used to evaluate the effects of DLP4 on bacterial membrane by flow cytometry. As shown in **Figure 3**, in the absence of peptide, the percentage of *S. hyicus* ACCC 61734 stained with PI was 0.080%. The PI-permeated percentages of cells treated with 1× MIC DLP4 were 0.403, 2.28, and 2.12% for 5, 30, and 120 min, respectively, and those of cells treated with 2×MIC DLP4 were 1.56, 2.95, and 1.93% for 5, 30 and 120 min, respectively, and those of cells treated with 4× MIC DLP4 were 1.93, 2.95 and 2.02% for 5, 30 and 120 min, respectively. The PI-permeated rate of DLP4 was similar with ceftriaxone (CRO) (≤2.84%). The positive control nisin displayed the strong PI-permeated rate, which was >35% even at 1× MIC for 5 min treatment. These results indicated that DLP4 has little effect on membrane integrity.

#### SEM and TEM Observations

**Figure 4:**
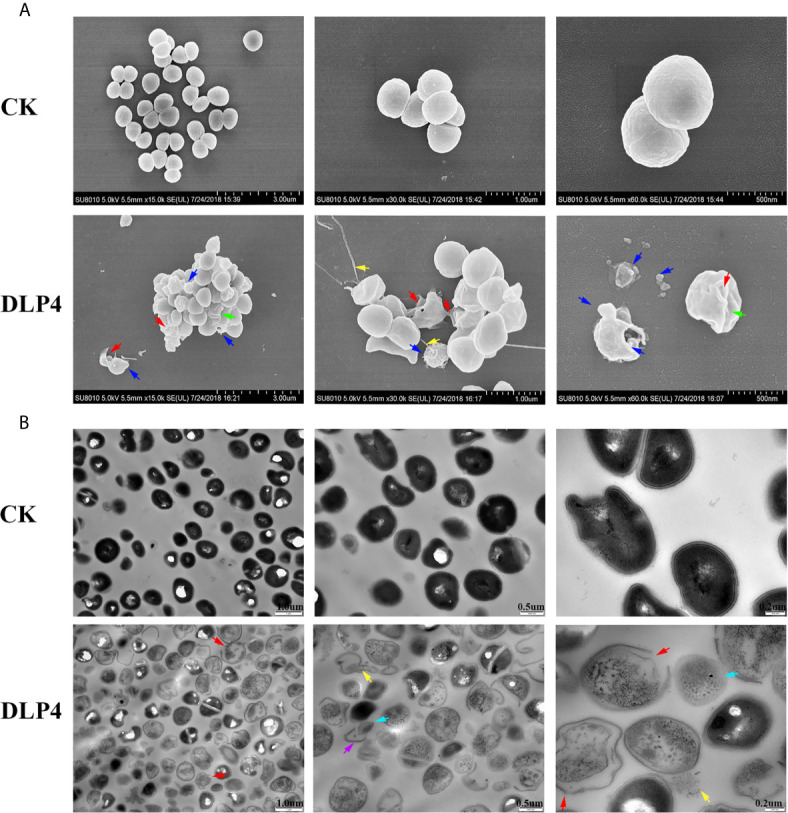
Observation of the effect of DLP4 on the cell membrane and morphology of *S. hyicus* ACCC 61734. **(A)** SEM images of *S. hyicus* ACCC 61734 treated with 4× MIC DLP4 for 2 h. **(B)** TEM images of *S. hyicus* ACCC 61734 treated with 4× MIC DLP4 for 2 h. The arrows indicate specific symptoms. Red arrow: membrane perforation; blue arrow: membrane vesicles; yellow arrow: leakage of cytosol; green arrow: cell shrinkage; purple arrow: cell wall; cyan arrow: ghost cell.

SEM observations showed that the untreated *S. hyicus* ACCC 61734 cells were round and undamaged. While after incubation with 4× MIC DLP4, holes were observed in the bacterial peptidoglycan layer (cell wall), membrane vesicles were released resulting in the leakage of cellular contents. Moreover, amounts of shrinking cells and filiferous adhesions could also be observed (**Figure 4A**). The effect of DLP4 on *S. hyicus* ACCC 61734 was also visualized by TEM. As shown in **Figure 4B**, the untreated cells had intact membrane and their cytoplasm electron density appeared to be homogeneous. However, after treatment with DLP4, considerable changes were observed in cell morphology, the invagination of intracellular membrane, cell wall disruption and leakage of cellular content. Besides, the electron density became heterogeneous and a lot of ghost cells appeared in *S. hyicus* (approximately 95%) (**Figure 4B**).

### Efficacy of DLP4 in a Mouse Model of Cutaneous Abscesses

To verify the antibacterial efficacy of DLP4 on local infections, mice were subcutaneously infected with *S. hyicus* ACCC 61734 and followed by the treatment with DLP4, the efficacy of DLP4 was measured as described below.

#### Observation of Abscess Symptoms

**Figure 5:**
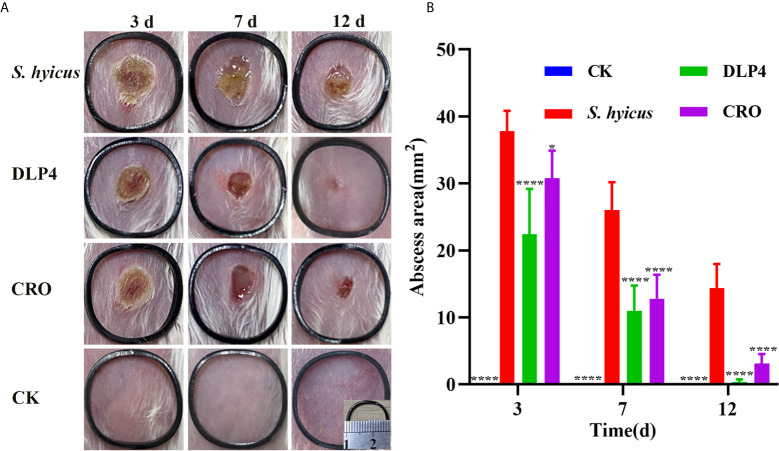
The therapeutic effect of DLP4 in mouse model of cutaneous abscesses induced by *S. hyicus* ACCC 61734. Six weeks BALB/c mice were subcutaneously injected with *S. hyicus* ACCC 61734, and followed by the treatment with DLP4 and ceftriaxone (CRO) at 1 d after infection. **(A)** Photographs of abscess at days 3, 7, and 12 after challenge with *S. hyicus* ACCC 61734, the length and width of the black ring are 1.6 cm. **(B)** The abscess area of different groups, results were given as mean ± SD (n = 5), all data were analyzed by the one-way ANOVA and Bonferroni multiple comparison, *P < 0.05; ****P < 0.0001.

The abscess symptom was monitored over the period of 12 d treatment and the abscess area was measured at 3, 7, and 12 d. As shown in **Figure 5A**, during the entire healing period, DLP4 and ceftriaxone treatment all significantly reduced the scope of abscess and alleviated the symptom, especially the DLP4 treatment group, which showed no significant difference from that of the blank control group, while the negative control group showed limited self-recovery. The therapeutic effect of DLP4 and ceftriaxone were further illustrated by the size of abscess area, as shown in **Figure 5B**, at 3, 7, and 12 d, the average abscess areas of DLP4-treated mice were 22.4, 11, and 0.35 mm^2^, respectively, and ceftriaxone-treated mice were 30.8, 12.8, and 3.1 mm^2^, respectively, which were significantly smaller than those of the negative group (37.8, 26, and 14.4 mm^2^, respectively).

#### Inhibition of Bacterial Translocation

**Figure 6:**
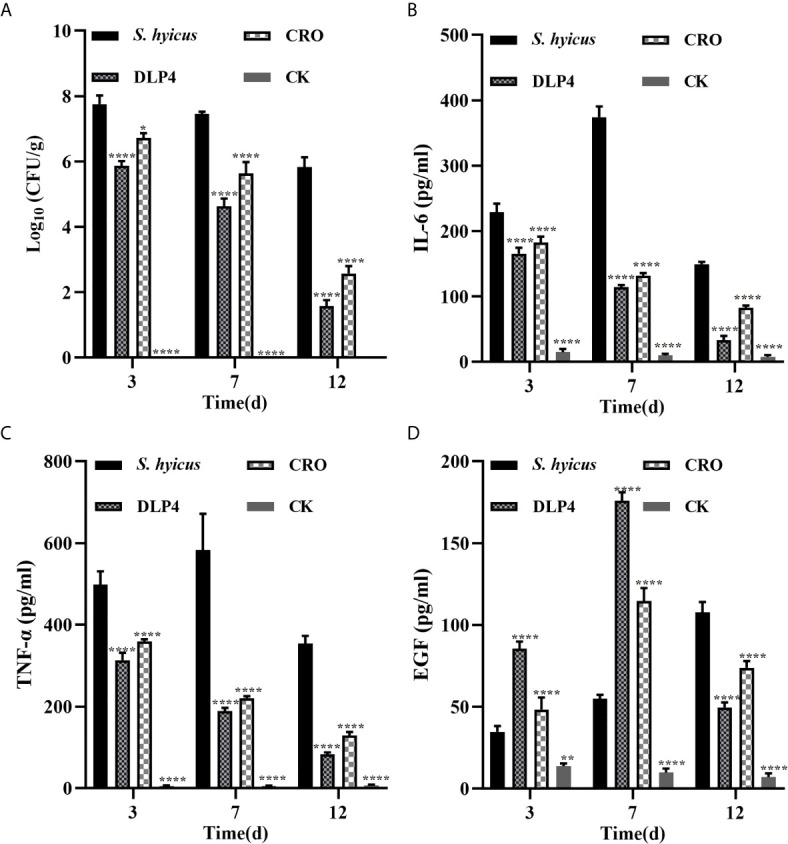
The therapeutic effect of DLP4 in mouse model of cutaneous abscesses induced by *S. hyicus* ACCC 61734. **(A)** The effect of DLP4 or ceftriaxone (CRO) on bacterial burdens of cutaneous abscesses in *S. hyicus*-infected mice. Data are presented as mean ± SD. **(B–D)** The regulatory effect of DLP4 or CRO on inflammatory factors **(B)** TNF-α **(C)** IL-6 and **(D)** epidermal growth factor (EGF). All data were analyzed by the one-way ANOVA and Bonferroni multiple comparison. *p < 0.05, **p < 0.01, ****p < 0.0001.

As a crucial evaluation of DLP4 efficacy, the bacterial burden of each abscess was determined. As shown in **Figure 6A**, compared with negative control group, the amount of bacterial in the abscesses of the DLP4 and ceftriaxone treated mice were significantly decreased, they were particularly manifested as the bacterial load of DLP4 treatment groups decreased by 24.3, 37.9, and 73.1% at days 3, 7, and 12, respectively, which was slightly superior to ceftriaxone treatment group (13.4, 24.4, and 55.8%, respectively). The significant reduction of bacteria in groups treated with DLP4 and ceftriaxone were consistent with the results of the reduction in abscess area (**Figure 5**).

#### Regulation of Cytokines

To further investigate whether the protective effect of DLP4 was related to the regulation of inflammatory factors and epidermal growth factor, the skin lesions were collected to measure the levels of IL-6, TNF-α, and EGF. As shown in **Figures 6B, C**, compared with the untreated group, the levels of IL-6 and TNF-α in DLP4-treated group decreased at 27.7, 69.4, 78.0, 37.2, 67.6, and 76.7% at days 3, 7, and 12, respectively, which showed no significant difference compared with the ceftriaxone-treated group (20.3, 64.8, 44.7, 28.0, 62.1, and 63.5% at days 3, 7, and 12, respectively). Furthermore, compared with the untreated group, the expression of the EGF in DLP4-treated mice (146.7 and 220.7%, respectively) and ceftriaxone-treated mice (39.6 and 108.9%, respectively) prominently increased in the early stage (3 and 7 d) but reduced quickly over time (54.0 and 31.5%, 12 d, respectively) (**Figure 6D**).

#### Histopathology Analysis

**Figure 7:**
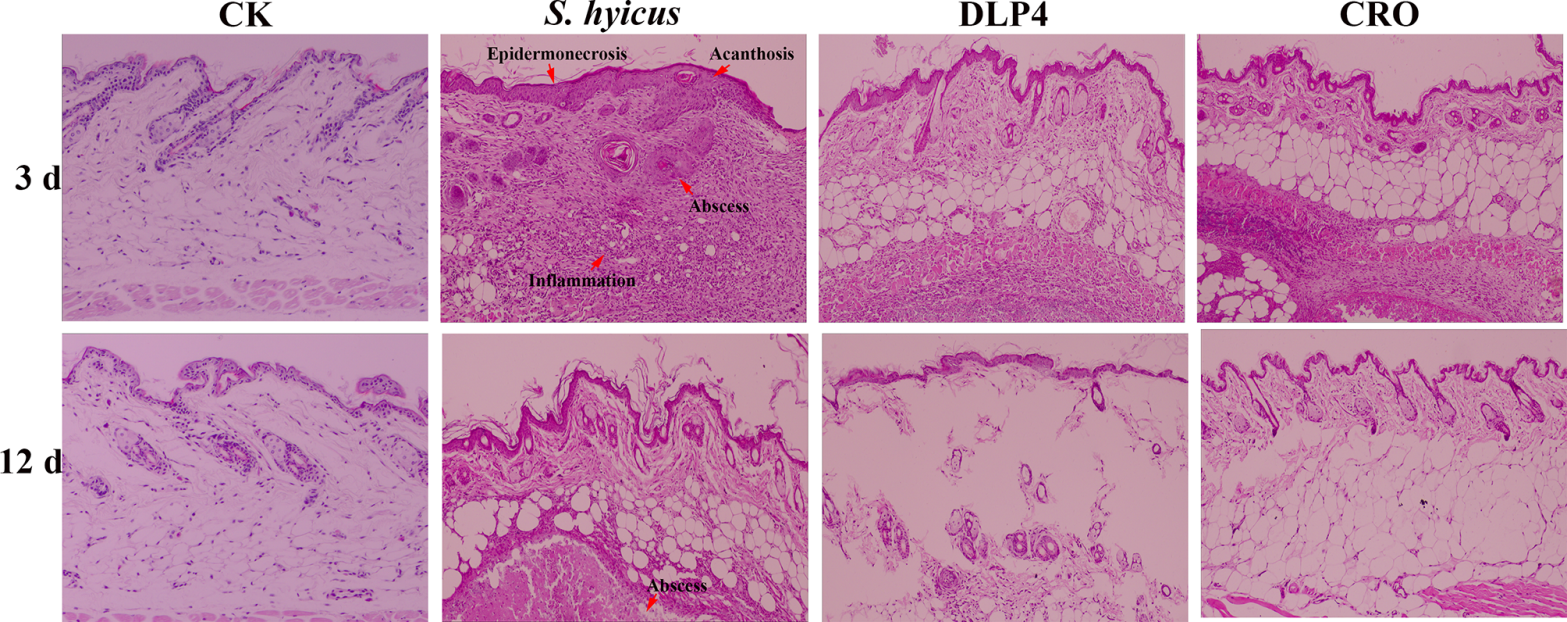
The protective effect of DLP4 on mice skin lesion induced by *S. hyicus* ACCC 61734. Histological assays of skin abscesses from mice (magnifications, ×100) at 3 and 12 d. CK: the uninfected mice; *S. hyicus*: the infected mice without treatment; and treatment groups with DLP4 and ceftriaxone (CRO), respectively.

Histopathological assays of the abscess tissues were operated at 3, 12 d after infection with *S. hyicus.* As shown in **Figure 7**, skin lesions from negative-treated mice exhibited as: extensive inflammatory cells infiltrated, skin stratum spinosum was significantly thickened and keratinized, the dermis showed obvious purulent inflammatory necrotic lesions. Abscess symptoms gradually recovered with the time extension. Whereas, DLP4-treated and ceftriaxone-treated mice displayed relatively moderate epidermal necrosis and cellulitis, which indicated that the mice treatment with DLP4 and ceftriaxone recovered rapidly.

## Discussion

In recent years, there is a great concern about the problem of bacterial resistance to antibiotics, which used in human medicine as well as in veterinary. Therefore, it is urgent to develop antibiotic alternatives to combat bacterial resistance. The prevention and control of *S. hyicus* infection is facing same problems (Park et al., 2013; Park et al., 2018). AMPs have shown great promise because they cause bacteria to develop no/low resistance. AMPs are produced by many organisms ranging from bacteria to fungi, plants, and animals, in particular, insect defensins have been considered as promising antibiotic alternatives, and their researches are getting more and more attention. In this study, the antibacterial activities, pharmacological characteristics, and *in vitro* mechanism of DLP4 against *S. hyicus* were systematically investigated.

Previous studies have shown that DLP4 exhibited a broad spectrum and high-efficiency bactericidal activity against Gram-positive bacteria, including *S. aureus*, *S. epidermidis*, *S. suis* and *S. hyicus*, which can effectively prevent secondary infections of environmental microorganisms on the basis of skin damage (Park et al., 2015; Li et al., 2017) (**Table 1**). PAE is an important indicator of efficacy evaluation, reflecting the inhibitory effect of the drug on bacteria after a brief contact with bacteria (Bundtzen et al., 1981; Davoodi et al., 2020). In this study, the PAE of DLP4 against *S. hyicus* ACCC 61734 was almost 10 times than that of ceftriaxone at 2× MIC (**Figure 1B**), which may prolong the administration interval, reduce medical costs, and delay the occurrence of drug resistance. This study also proved that under long-term exposure to DLP4 at a subinhibitory concentration, *S. hyicus* ACCC 61734 was unlikely to develop drug resistance (**Figure 1C**), which further proved that DLP4 presented no/low resistance pharmacodynamic properties. Besides, DLP4 displayed a synergistic activity with β-lactam antibiotics (ceftriaxone, penicillin, and amoxicillin) against multi-drug resistant *S. hyicus* (**Table 2**). Ceftriaxone sodium, penicillin and amoxicillin inhibit the synthesis of bacterial cell wall by inhibiting D-alanyl-D-alanine transpeptidase, leading to bacterial death, which is the bactericide of breeding period, and ofloxacin leads to bacterial death by inhibiting the activity of bacterial DNA gyrase enzyme, preventing the synthesis and replication of bacterial DNA (Alves-Barroco et al., 2020). DLP4 and NZ2114 have the same tertiary structure, and belong to CSαβ defensins, and the action of DLP4 and NZ2114 was on cell wall (Li et al., 2017). Study has indicated that plectasin NZ2114 was synergistic in combination with cell wall targeting antibiotics (teicoplanin, moenomycin, and dalbavancin) (Breidenstein et al., 2015), which provides an evidence for the synergistic effect of DLP4 and β-lactam antibiotics. The synergistic effects of DLP4 combination with antibiotics pave a novel way for the commercial application of insect defensin. The combination of insect defensins and antibiotics could restore sensitivity of multi-drug-resistant pathogens, which will help to reduce the use of antibiotics and then delay the development of drug resistance (Dhand and Sakoulas, 2014; Mylonakis et al., 2016; Lazzaro et al., 2020).

Previous studies have shown that *S. aureus* can invade eukaryotic cells and evade the bactericidal action of conventional antibiotics (Wang et al., 2018a; Wang et al., 2018b; Li et al., 2020). This is the first study to prove that *S. hyicus* ACCC 61734 can be internalized by HaCaT cells, which help *S. hyicus* to evade conventional antibiotic killing (**Figure 2**). The corresponding conclusion was also obtained through this experiment, almost ceftriaxone does not have the ability to enter into cell, which was consistent with previous study (Prakash et al., 2013), which is due to its hydrophilicity, resulting in low cell penetration (Lipinski et al., 2001; Zaki and Hafez, 2012; Prakash et al., 2013). Since DLP4 and NZ2114 have the same molecular structure CSαβ, we can reasonably to speculate that they share the same internalization mechanism: clathrin-mediated and polypinocytosis promote the accumulation of intracellular antimicrobial peptides and achieve effective sterilization (Wang et al., 2018b). It was further certified in this study: after treatment with DLP4, almost 99.9% intracellular *S. hyicus* ACCC 61734 were killed, which was similar to previous study (**Figure 2**) (Li et al., 2020).

It has been proved that the mode of action of insect AMPs mainly focus on the interaction between AMP and bacterial cell membrane. This is mainly based on the cationic and hydrophobic characteristics, which make the insect AMP electrostatically attracted to the bacterial cell membrane, to establish connection, and further lead to the accumulation of hydrophobic residues, causing the outer leaves of the cell wall to swell and thin, and eventually form holes or even cause bacterial dissolution (Brown and Hancock, 2006; Mylonakis et al., 2016; Haney et al., 2017; Wang et al., 2021). In this study, the impacts of DLP4 on cell membrane were determined with FACS, SEM and TEM. The results indicated that the penetration rates of the DLP4 on membrane were controlled within 4% and did not have time and dose dependence, which is in consistent with previous study (**Figure 3**) (Li et al., 2017), and SEM and TEM images displayed amounts of shrinking cells, invagination of intracellular membrane and a lot of ghost cells. This phenomenon may be explained by the “bubbling cell death” triggered by DLP4, which manifested by DLP4 weakening cell wall, forming holes in the peptidoglycan, stimulating the formation of cytoplasmic vesicles, and thus leading to release of the membrane vesicles and form ghost cells (**Figure 4A**) (Toyofuku et al., 2019). While the underlying molecular mechanism is not clear, which may be related to conservative residues Asp4 and Arg23, which need to be further proved. (Brogden, 2005; Cerovsky et al., 2011; Li et al., 2017).

Previous studies have indicated that AMPs not only have high-efficiency bactericidal functions, but also have immunoregulatory function, such as LL-37 and HBD2 (Funderburg et al., 2007; Semple et al., 2011; Tewary et al., 2013). To explore the immunomodulatory activities of DLP4, a mouse abscess model was established, as the healing of skin abscess is a complex, coordinated and orderly process to promote tissue repair and regenerate, there are a variety of cells and cytokines participate in the process (Rousselle et al., 2018; Hou et al., 2020). In this study, after challenged with *S. hyicus* ACCC 61734, humoral immunity and celluar immunity were rapidly activated to protect mice from fatal injury. After treatment with DLP4, the levels of TNF-α, IL-6 decreased (**Figures 6B, C** **)**, the level of EGF significantly increased and followed by a decreased with the extension of treatment time (**Figure 6D**). TNF-α, as the initiator of tissue repair, is a potent stimulator of chemotaxis and degradative responses required for wound healing, and it can accelerate the wound healing. When infected by *S. hyicus* ACCC 61734, macrophages, mast cells and keratinocytes were activated, which can further promote the differentiation of T cells and the release of TNF-α and IL-6, further attract and activate neutrophils and monocytes, inducing fibroblasts to produce prostaglandin E (PGE) and collagenase. Simultaneously, EGF is a mitotic stimulator. After binding to receptors on the cell membrane of skin tissues, EGF induces or directly regulates important genes for cell proliferation through autophosphorylation, and stimulates the division and proliferation of damaged cells, further initiates wound repair, and strongly promotes the growth of epithelial cells effect (Schultz et al., 2004). Consistent with this, DLP4 possesses substantial wound healing properties in mice, which may be based on its ability to balance cytokine release, while the immune regulation and function need to be further confirmed through cell signaling pathway (Mylonakis et al., 2016).

## Conclusion

In this study, *in vitro* and *in vivo* multiple effects of DLP4 against *S. hyicus* ACCC 61734 were systematically investigated. DLP4 displayed excellent antibacterial and pharmacological activity and killed *S. hyicus* ACCC 61734 cells by destroying cell wall and generating membrane vesicles. *In vivo* experimental results showed that DLP4 displayed potent therapeutic effect on mouse abscess by inhibiting bacterial proliferation and regulating cytokines. All results provide evidence that DLP4 may be used as promising drug for the treatment of EE caused by *S. hyicus* ACCC 61734.

## Data Availability Statement

The original contributions presented in the study are included in the article/supplementary material. Further inquiries can be directed to the corresponding authors.

## Ethics Statement

The animal study was reviewed and approved by the Laboratory Animal Ethical Committee and its Inspection of the Feed Research Institute of CAAS (AEC-CAAS-20090609).

## Author Contributions

XM, NY, and DT: conception and experiments design. XM: experiments operation. XM, NY, RM, and YH: methodology and data analysis. XM: writing—original draft. JW, NY, and DT: writing—review and editing. JW and XY: contributed in funding acquisition. All authors contributed to the article and approved the submitted version.

## Funding

This study was supported by the National Natural Science Foundation of China (No. 31872393), the Agricultural Science and Technology Innovation Program (ASTIP) in CAAS (CAAS-ZDXT2018008 and CAAS-ASTIP-2013-FRI-02), and grant NH2021202202 of the Quality Control for Feed and Products of Livestock and Poultry Key Laboratory of Sichuan Province.

## Conflict of Interest

Author XY was employed by New hope Liuhe Co., Ltd.

The remaining authors declare that the research was conducted in the absence of any commercial or financial relationships that could be construed as a potential conflict of interest.
